# Geographic and Habitat Origin Influence Biomass Production and Storage Translocation in the Clonal Plant *Aegopodium podagraria*

**DOI:** 10.1371/journal.pone.0085407

**Published:** 2014-01-10

**Authors:** Tina D′Hertefeldt, Johanna M. Eneström, Lars B. Pettersson

**Affiliations:** Biodiversity Unit, Department of Biology, Lund University, Lund, Sweden; Beijing Forestry University, China

## Abstract

Through physiological integration, clonal plants can support ramets in unfavourable patches, exploit heterogeneously distributed resources and distribute resources that are taken up over large areas. Physiological integration generally increases in adverse conditions, but it is not well known which factors determine the evolution of physiological integration. The aim of this study was to investigate if clonal plants from Southern and Northern populations of the clonal herb *Aegopodium podagraria* differed in physiological integration in terms of translocation of carbon to the rhizomes, and in biomass production using a reciprocal transplant experiment. *Aegopodium podagraria* from shaded conditions have been suggested to share more resources than clones from open conditions and therefore, plants from forest and open populations within the Southern and Northern regions were included. The regional growing conditions greatly affected biomass production. Plants grown in North Sweden produced more biomass and allocated more biomass to shoots, while plants grown in South Sweden allocated more biomass to rhizomes. There was a regional origin effect as plants originating from North Sweden produced more biomass in both regions. Within the Northern region, plants from shaded habitats translocated more ^14^C to the rhizomes, suggesting more storage there than in plants from open habitats. In addition to genetic differentiation in biomass production between Northern and Southern populations, probably as a response to a shorter growing season in the North, there appeared to be genetic differentiation in physiological integration within the Northern region. This shows that both regional and local conditions need to be taken into account in future studies of genetic differentiation of physiological integration in clonal plants.

## Introduction

Clonal plants are successful in many habitats, from harsh tundra sites to being invasive species in highly productive habitats [1], [2]. The ability to support parts of a clone in an unfavourable patch by means of physiological integration has been put forward as one trait that contributes to the success of clonal plants [2]–[6]. In the same way that physiological integration permits clonal plants to deal with spatially heterogeneous resource supply, storage permits plants to counteract temporally heterogeneous resources [7], [8]. In general, plants that experience a time lag between resource supply and resource demand show greater dependence on resource storage than plants where resources are used immediately for growth [8], [9]. Plants can store resources in three ways: accumulation, reserve formation and recycling [9]. Resources that go into reserve formation are synthesized into storage compounds (such as starch) instead of going directly into growth. This type of storage competes for resources with growth and reproduction. For plants in temperate regions, stores are created at the end of the growing season and stored for later use, creating a time lag between the creation of stored resources and the use of them at a later time. In *Aegopodium*, stored resources are used for rapid growth in early spring [7]. In northern regions, the growing period is shorter and the unfavorable winter period is longer. There will therefore be a longer time lag between creation and use of stored resources in more northern regions than in southern regions.

Many plants have been seen to expand North following present temperature increases [10], and plant species that grow at higher latitudes have lower biomass [11]–[13] and higher allocation to storage organs at higher latitudes or altitudes [14], [15]. In fish, higher storage rates were found in Northern populations than in Southern populations, while the amount of stored reserves did not differ [16]. Populations that expand Northwards would experience gradually increased time lags between resource supply and demand which might lead to lower allocation to biomass and higher allocation to storage.

However, in response to latitudinal gradients, plants have been shown to increase their growth rates to compensate for a shorter growing season [17], [18]. In a study that specifically targeted the length of the growing season, plants had lower production when the growing season at the original habitat of the population was longer [15]. The ability of high latitude populations to compensate for short growing periods has been demonstrated for a range of organisms [17], [19]. Apart from changing growth rate, perennial plants may increase stored reserves when conditions get harsher or when the period between supply and demand is long. Plants that expand their range Northwards could therefore either change their biomass production or change the allocation pattern towards more storage. In fact, two forest herbs showed divergent responses along a latitudinal gradient in terms of growth and reproduction, experiencing either benefits of a higher temperature or suffering the costs of lower light availability at lower latitudes [20]. In order to investigate plant responses to a more northerly growing site, integrated studies should take into account both biomass production and allocation to storage.

As seen from the study of De Frenne et al. [20], a plant's response to a latitudinal gradient will depend on its life-history strategy. In the case of fast-growing, weedy plants, it is known that they utilize high resource levels by high relative growth rates [21]. In clonal plants, which are common at Northern latitudes, resources are stored by translocating carbohydrates to rhizomes and other storage organs to be used at the start of the growing season [1], [2], [22]. Through physiological integration, resources are shared among ramets, and plants of arctic, subarctic and forest habitats have been found to have high degrees of physiological integration [23]–[28]. Resource translocation can therefore be assumed to be important for clonal plants that grow at northern latitudes.

One productive plant that is found at northern latitudes is ground elder, *Aegopodium podagraria*, a clonal herb which has been expanding northwards in Scandinavia during the last 40 years and is an invasive species in North America, where it has been introduced [29]–[33]. *Aegopodium* occurs in open as well as shaded habitats up to high latitudes in Scandinavia. It relies on stored carbohydrates in rhizomes for rapid development of above-ground parts after the winter [7]. Subsidising ramet establishment in unfavourable patches via physiological integration is a trait that increases the invasiveness of clonal species [2], and the storage of carbohydrates in rhizomes is likely to have contributed to the success of invasive clonal weeds such as *Fallopia japonica* [22]. Stored resources and the subsequent utilization of them via physiological integration may therefore be an important trait enabling *Aegopodium* to colonize the range of habitats it is found in. In addition, forest clones of *Aegopodium* have previously been found to be more dependent on resource sharing than clones from open habitats [5]. In the present study, growth and allocation to storage was investigated in a Northern and a Southern region along a climatic gradient in *Aegopodium*, including populations from open and shaded habitats in each region.

In a reciprocal transplant experiment we compared biomass and storage patterns in plants from North Sweden and South Sweden. The Northern region is harsher during winter and has a shorter and cooler growing period, suggesting that storage should be larger during the longer winter than further South. We hypothesized i) that all plants would produce a lower biomass when placed in the North, and ii) that plants originating from North Sweden would produce less biomass at both sites than plants from South Sweden. Resource storage is assumed to be larger when the time between resource acquisition and resource use is longer [9], and we therefore investigated if iii) plants grown in North Sweden allocated more carbohydrates to storage than plants grown in South Sweden and iv) if plants of a Northern origin allocated more carbon to storage than plants of a Southern origin. Since seasonal variation in light is more pronounced in shaded habitats than in open habitats, we hypothesized that v) plants from deciduous forest habitats would allocate more carbon to storage than plants from open habitats.

## Materials and Methods

### Study areas and species

The plant material was collected in a Southern (Skåne) and a Northern (Västerbotten) region, 1200 km apart (Table 1). No specific permissions were required for the collections of plant material since this is not a threatened species. It is very common and regarded as a weed in Sweden. Access to the land was granted according to the Right of Public Access in Sweden. *Aegopodium podagraria* is a clonal herb that spreads vegetatively by hypogeogenous rhizomes (rhizomes originating below ground, typically with fast vegetative spread) [34]. The rhizome connections between consecutive ramets are more than 10 cm long. Clones consist of several ramets which have the potential to share resources among them [33]. The compound leaves of *Aegopodium* become more complex when the plant develops from juvenile to an adult stage [35]. The small, white, five-petaled flowers are organized in compound umbels of 280–1400 flowers, and the shoots are up to 1 m tall [36]. The rhizome system is strong-growing and can consist of tens of ramets forming branched clonal systems of many meters (Eneström pers. obs.).

**Table 1:** *Aegopodium podagraria* populations collected in North Sweden (Västerbotten) and South Sweden (Skåne).

Region	Population	Habitat	Latitude N	Longitude E	Temp	Precipitation	PAR
Västerbotten	Kylören	Open	62° 32′ 13′′	19° 46′ 0′′	4.4±0.3	642±27	6530±70
Västerbotten	Gubböle	Open	63° 54′ 4′′	19° 53′ 16′′	3.4±0.3	710±28	6060±90
Västerbotten	Västerbacken	Open	63° 42′ 23′′	20° 20′ 57′′	4.4±0.3	603±24	6340±80
Västerbotten	Norrbyn	Open	63° 33′ 52′′	19° 49′ 20′′	4.5±0.3	618±26	6510±70
Västerbotten	Djupviksgatan	Open	63° 42′ 41′′	20° 22′ 21′′	4.3±0.3	607±25	6360±70
Västerbotten	Norrfors	Shaded	63° 33’ 52′′	19° 49′ 20′′	4.5±0.3	618±26	6510±70
Västerbotten	Kylören	Shaded	63° 32′ 13′′	19° 46′ 0′′	4.4±0.3	642±27	6530±70
Skåne	Vedhygge	Open	56° 6′ 37′′	13° 51′ 8′′	8.2±0.2	791±34	6990±140
Skåne	Kristianstad	Open	56° 0′ 54′′	14° 11′ 54′′	8.3±0.2	656±33	7260±130
Skåne	Högevall	Open	55° 41′ 52′′	13° 10′ 57′′	9.0±0.2	785±34	6690±140
Skåne	Johanneslust	Open	55° 36′ 11′′	13° 2′ 44′′	9.2±0.2	671±32	7230±130
Skåne	Revinge	Open	55° 42′ 52′′	13° 30′ 25′′	8.8±0.2	769±32	7140±130
Skåne	Plantholmen	Shaded	55° 42′ 17′′	13° 34′ 56′′	8.7±0.2	739±32	7160±130
Skåne	Öveds Eke	Shaded	55° 41′ 2′′	13° 32′ 56′′	8.7±0.2	728±31	7150±130
Skåne	Kulleberga	Shaded	55° 52′ 16′′	13° 28′ 14′′	8.3±0.2	803±33	7070±150
Skåne	Oretorp	Shaded	56° 6′ 4′′	13° 51′ 31′′	8.2±0.2	791±34	7000±140
Skåne	Ignaberga	Shaded	56° 6′ 37′′	13° 51′ 31′′	8.2±0.2	768±34	6990±140

*Aegopodium podagraria* populations collected in North Sweden (Västerbotten) and South Sweden (Skåne). Coordinates are given in the Swedish grid system (RT90 2.5 gon V). The regions were 1200 km. apart. The habitats were either open or shaded (forest). Yearly data (mean ± SE) for temperature (Temp), precipitation and photosynthetically active radiation (PAR, given as photosynthetic photon density [mol m^−2^]) during 2002–2011 were obtained from the meteorological databases STRÅNG [37] and PTHBV [38].

The North has a shorter growing season, lower annual temperature with more frost days, lower precipitation and more sunlight hours (Tables 1 and 2). During the growing period of the experiment (May to September), the total number of sun hours was higher in the North than in the South (Umeå: 1114 h, Lund, 987 h) [37] (Table 2) whereas photosynthetically active radiation (PAR) was higher in the South than in the North (Lund: 4592 mol m^−2^; Umeå 4379 mol m^−2^) [37] (Table 2). The average temperature was higher in the South than in the North (Lund: 16.9°C; Umeå: 14.3°C) [38] (Table 2). Precipitation during the experiment was 388 mm in the South and 203 mm in the North [38] (Table 2). All these differences except PAR are significant over the period 2002–2011 (paired t-tests: sun hours *P* = 0.0145; PAR *P* = 0.12; temperature *P*<0.001; precipitation *P* = 0.032, Table 2).

**Table 2:** Climate data for the experimental gardens.

**Experimental garden**	**Temperature**	**Precipitation**	**PAR**	**Sun hours**	**Growing season**
Umeå	14.3 (13.6±0.3)	203 (274±18)	4379 (4266±93)	1114 (1119±46)	160
Lund	16.9 (16.1±0.2)	388 (354±33)	4592 (4487±145)	987 (923±57)	210

Climate data for the two experimental gardens. Temperature is given as the average during the course of the experiment (Umeå: 11 May to 14 September 2006; Lund: 9 May to 21 September 2006) and the yearly average ± SE for the corresponding periods in 2002–2011. Precipitation, photosynthetically active radiation (PAR, given as photosynthetic photon density [mol m^−2^]) and sun hours are given as sums during the course of the experiment and yearly sums SE for the corresponding periods in 2002–2011. Growing season (days) in the two areas and all other data were obtained from the meteorological databases STRÅNG [37] and PTHBV [38].

In each region, *Aegopodium* rhizome fragments with at least one visible bud were collected from five open and five (Southern region) or two (Northern region) shaded habitats, in total 17 habitats (Table 1). To avoid collection of rhizomes from the same clone, only one rhizome fragment was collected at each site. One rhizome fragment was collected at each site in order to sample several sites in each region. The open habitats were roadsides or gardens, and the shaded habitats were mixed deciduous forests. In North Sweden, natural deciduous forests were scarce because the vegetation period is too short for many of the deciduous trees that make up the forests where *Aegopodium* is found [39]. At one of the sites, *Aegopodium* may have been introduced by ballast dumping, and botanical records list *Aegopodium* as being present at the site already in 1928 [40]. This site, Kylören, is a deciduous forest with bird cherry (*Prunus padus*), grey alder (*Alnus incana*), rowan (*Sorbus aucuparia*) and other trees and a vegetation with among other herbs *Arenaria serpyllifolia*, *Geum urbanum*, *Potentilla crantzii* and *Medicago lupulina*. All sites where *Aegopodium* was collected were nutrient rich and among other species were *Urtica dioica*, *Mercurialis perennis, Dactylis perennis* and *Geum urbanum*.

The rhizome fragments were multiplied clonally in the greenhouse at Lund University using standard protocols [5] until the start of the experiment. The rhizome fragments were collected in the field in 2004 and early 2005 and grown for at least 13 months (mostly 19) in the greenhouse, which resulted in the production of at least seven new, consecutive ramet generations. Each rhizome fragment produced several rhizome branches. Along each of these, the ten ramets used in the experiment were cut off, always with at least seven ramets produced in the greenhouse before any ramet was used for the experiment. This procedure was used to avoid maternal effects, and therefore only the youngest ramets were used in the experiment [33], [41]. Since *Aegopodium* is fast-growing, each rhizome fragment produced more than the 10 young ramets that were used in the experiment.

### Experimental set-up

Each experimental ramet consisted of a piece of rhizome with one leaf. The ramets used in the experiment were newly produced in the greenhouse. At planting, rhizome volume and root length of each replicate plant were measured using digital callipers and rulers and above-ground biomass was standardized to one leaf per plant.

There were no significant differences in size in the experimental material depending on regional or habitat origin, or between plants placed in Lund and Umeå (three-way ANOVA). Ten replicates were obtained from each clone, and each plant was planted in a five litre pot with regular potting compost (Kronmull™, Weibulls) mixed with natural washed sand. Half of the plants (5 replicates per clone) were placed in the experimental common garden belonging to Lund University, South Sweden on May 9, 2006. On May 11, 2006 the other plants were placed in an experimental common garden at Umeå University in North Sweden. The common garden sites were both unshaded and are called South and North to denote where the clones grew during the experiment. The origin groups are Open Northern, Shaded Northern, Open Southern and Shaded Southern. During the growing season the plants were checked regularly and were watered when needed, which was every second day during sunny weather.

### ^14^C labelling and analysis

At the end of the growing season, before plants in the North started to show signs of yellowing because of onset of autumn, one large leaf on each plant was labelled with ^14^C. The labelling was performed during the same week in September in Lund (19 September) and in Umeå (12 September). Labelling was performed by sealing a plastic bag supported by a wooden stick around each shoot. Then 1 ml of 2 µCi ml^−1^ bicarbonate solution was injected into a vial inside each plastic bag. CO_2_ was released by adding 1 ml 1M HCl into the vial and the injection hole was covered with sellotape to trap the ^14^C labelled CO_2_ inside the plastic bag [40]. The plants were left to photosynthesize and incorporate ^14^C for 1.5 hours at full sun light before the plastic bags were removed. Forty-eight hours after labelling, the plants were harvested, put in plastic bags and frozen in liquid nitrogen. They were then transported to the Department of Ecology in Lund and kept at −20°C until the roots were cleaned from soil and the plants were divided into four parts: labelled shoot, remaining shoots, rhizomes and roots. All plant parts were then dried to constant weight at 70°C and weighed. The different parts from each plant were ground separately and a sub-sample of 160–200 mg from each was combusted in a Packard Tri-carb Sample Oxidizer. The amount of ^14^C was measured in a Packard Tri-carb Scintillation Counter as disintegrations per minute (DPM). These values were used to calculate the mean percentage of ^14^C translocated to the different parts of the plants: labelled shoot, shoots, rhizomes and roots.

### Statistical analysis

A three-way ANOVA was used to analyse the effects of site (South or North), regional origin (Southern Sweden or Northern Sweden) and habitat origin (open or shaded habitat) on biomass production, biomass allocation and translocation of ^14^C. The analyses were performed on 1) dry weight of the entire plants and the different plant parts, 2) allocation of biomass to shoots, rhizomes and roots as percentage of total biomass and 3) partitioning of ^14^C activity (calculated as a percentage of ^14^C in labelled leaf, the rest of the shoots, rhizomes and roots compared to the amount of ^14^C calculated for the entire plant). When main effects were significant, differences between origin groups (Open Southern, Shaded Southern, Open Northern and Shaded Northern) were evaluated using 95% confidence intervals (CI). If needed, data were log transformed to improve variance homogeneity and normality to meet model assumptions [42]. All statistical analyses were carried out using SPSS 14.0 for Windows.

## Results

### Biomass production

**Figure 1:**
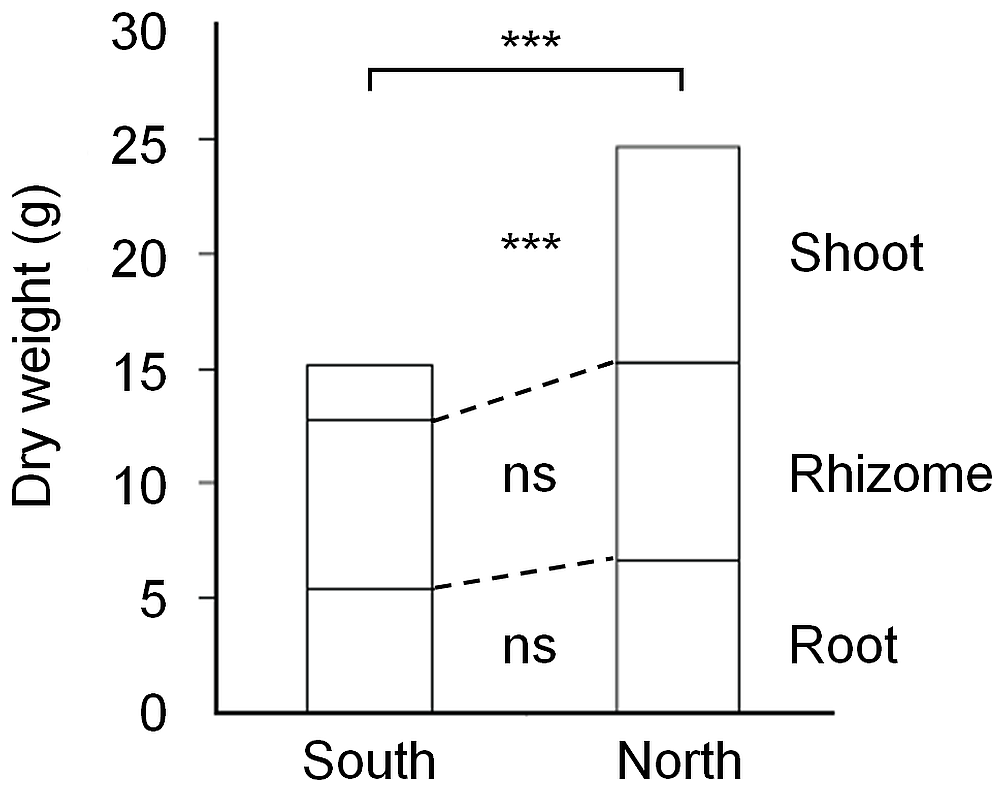
Biomass production of *Aegopodium podagraria* plants grown in South (Lund) and North (Umeå) Sweden. Significance levels: *** *P*<0.001, ns *P*≥0.05.

There were large differences in biomass production between plants grown in the South and plants grown in the North. Total biomass production was more than 1.5 times higher in the North (Figure 1, Table 3), due to a higher shoot production (*P* = 0.001). Rhizome and root biomass did not differ between sites (Figure 1). Shoots made up 41% of the biomass in the North compared to 16% in the South (*P*<0.001; Figure 1, Table 3), and accordingly, plants in the South allocated less biomass to shoots and more to rhizomes and roots than plants grown in the North.

**Table 3:** The effect of placement, region and habitat on biomass, biomass allocation and translocation of ^14^C in *Aegopodium podagraria* analysed by three-way Anovas.

Factor	Site (S)	Region (R)	Habitat (H)	R × H	S × H	S × R	S × R × H
Total biomass	16.602***	3.860*	0.708	3.200	0.115	0.278	0.020
Shoot biomass	176.630***	8.205**	1.021	1.542	1.541	0.975	0.007
Rhizome biomass	1.737	0.377	0.587	5.288*	0.012	0.561	0.093
Root biomass	1.270	0.919	0.075	0.036	0.424	0.215	0.252
Shoot biomass (%)	177.218***	0.341	0.327	1.17	1.967	0.042	0.015
Rhizome biomass (%)	33.473***	0.000	1.593	8.814**	0.904	0.009	0.001
Root biomass (%)	32.319***	0.316	1.091	7.500**	0.027	0.103	0.007
^14^C labelled leaf (%)	4.551*	0.373	0.145	5.148*	0.047	1.366	2.275
^14^C in shoot (%)	10.773***	0.559	1.864	0.876	2.053	0.640	1.137
^14^C in rhizome (%)	21.713***	2.599	3.619^1^	8.170**	0.152	0.487	0.953
^14^C in root (%)	2.535	0.926	1.827	1.658	0.002	0.064	0.039

The effect of placement, region and habitat on biomass, biomass allocation and translocation of ^14^C in *Aegopodium podagraria* analysed by three-way Anovas. Site is where the plants grew during the experiment, which was South or North Sweden. Regional origin (SSwe or NSwe) is denoted as region and habitat origin (shaded or open) as habitat. Biomass is measured as g dry weight. Degrees of freedom = 1 for all factors, total d.f. =  140. Numbers are F-values. Significant effects are denoted with asterisks; *** = *P*<0.001, ** = *P*<0.01, * = *P*<0.05, ^1^ *P* = 0.059.

**Figure 2:**
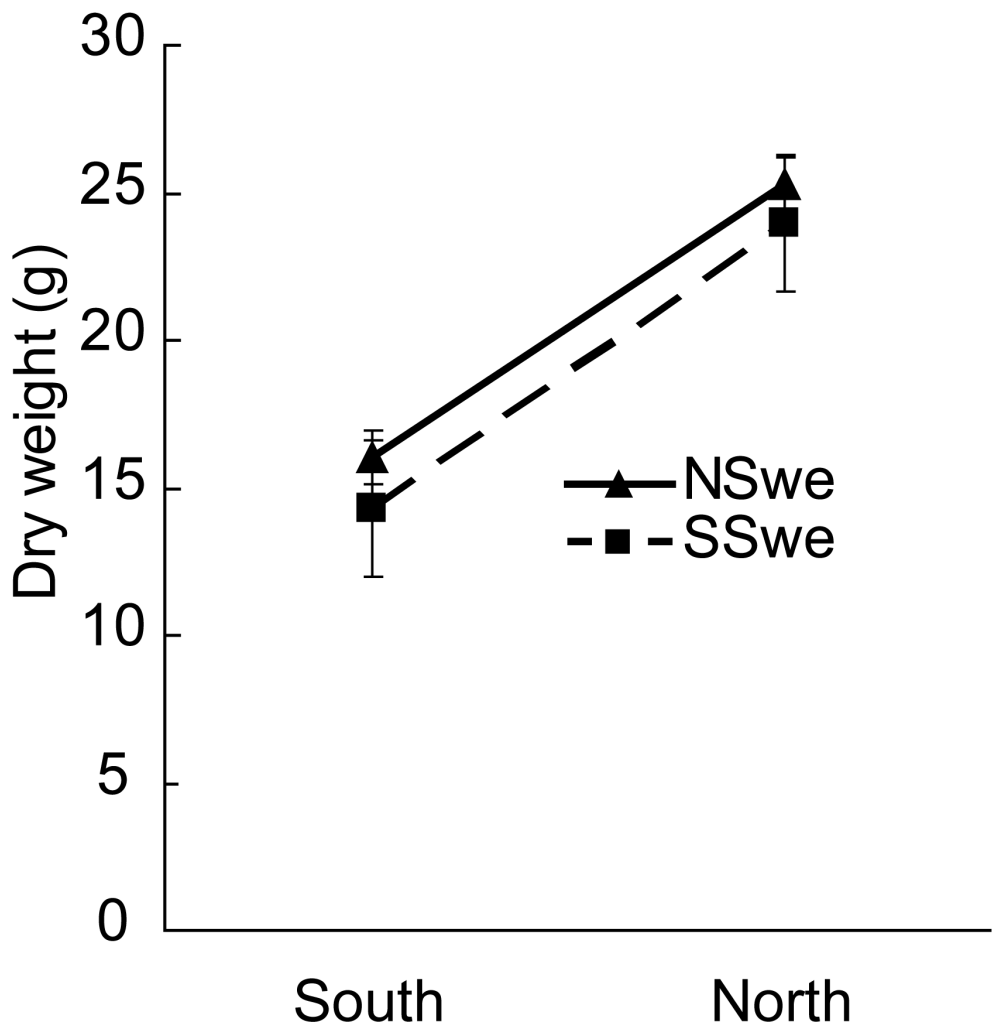
Biomass production in *Aegopodium podagraria* plants grown in South and North Sweden. The dry weight is g total plant biomass of shoots, rhizomes and roots. Clones originating from North Sweden (NSwe) produced more biomass both in their original region and in South Sweden (SSwe) (three-way Anova; region *P* = 0.05). South/SSwe: n = 44; South/NSwe: n = 35; North/SSwe: n = 35; North/NSwe n = 30.

Not only the growing site but also plant origin affected plant growth. Plants of the Northern origin produced more total biomass than plants of the Southern origin at both experimental gardens (*P* = 0.05; Figure 2, Table 3).

**Figure 3:**
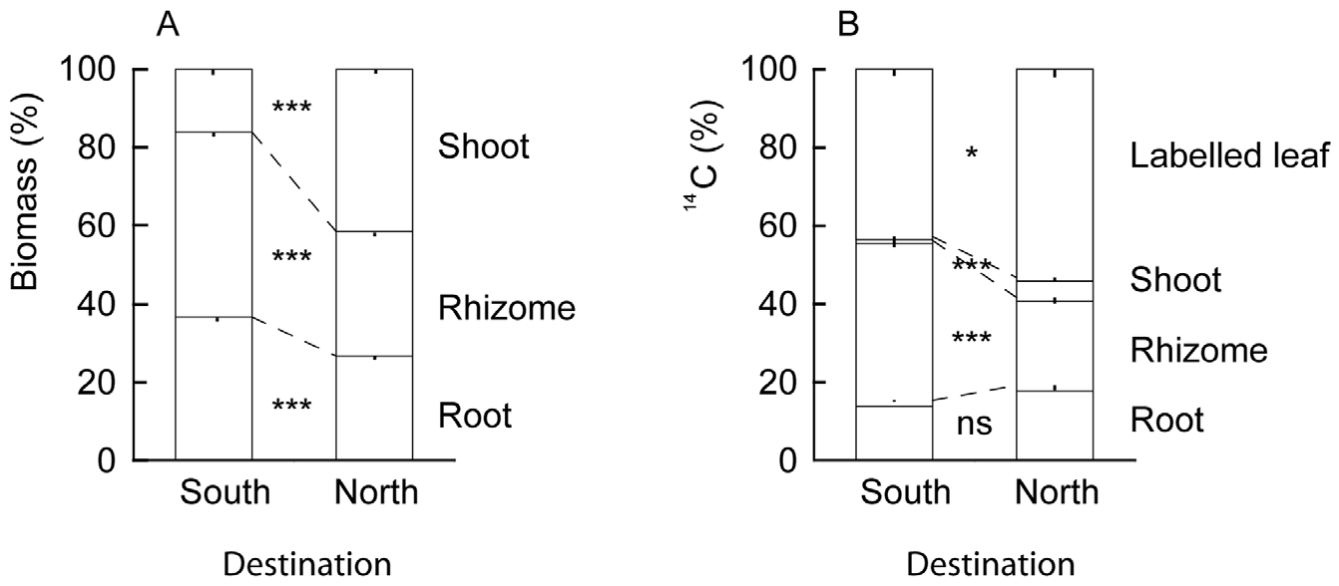
Allocation of biomass to shoot, root and rhizome of *Aegopodium podagraria* grown in South and North Sweden (Fig. 3a). Allocation of ^14^C to labelled leaf, shoot, rhizome and root of *Aegopodium* grown in South and North Sweden (Fig. 3b). Significance levels: *** = *P*<0.001, * = *P*<0.05. South: n = 75; North: n = 62.

**Figure 4:**
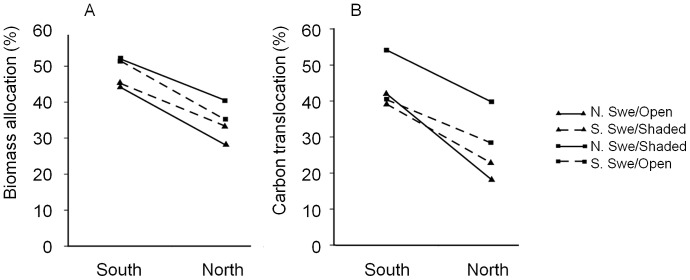
Allocation to rhizomes in plants grown in South and North Sweden, showing averages for the four origin groups (Fig. 4 a). Plants from SSwe/open habitats allocated more biomass to rhizomes than plants from NSwe/open habitats (*P*<0.05). Within the NSwe region, plants from shaded habitats tended to allocate more biomass to rhizomes than plants from open habitats (*P* = 0.055). Allocation of ^14^C to rhizomes in South and North Sweden, showing averages for the four origin groups (Fig. 4b). Plants from NSwe/shaded habitats translocated more ^14^C to the rhizomes than plants from NSwe/open (*P* = 0.05) and SSwe/shaded (*P* = 0.05) habitats. South/SSwe: n = 44; South/NSwe: n = 35; North/SSwe: N = 35; North/NSwe: N = 30.

The growing site significantly affected biomass allocation. Plants grown in the South allocated more biomass to rhizomes and roots, while plants grown in the North allocated more to the shoots (Figure 3a, Table 3). Biomass allocation to rhizomes differed depending on regional origin and habitat (regional origin × habitat, *P* = 0.023; Table 3). In the plants from open habitats, Southern plants allocated more biomass to rhizomes than Northern plants (*P*<0.05; Figure 4a). Within the Northern region, plants from shaded habitats tended to allocate more biomass to rhizomes than plants from open habitats (*P* = 0.055, Figure 4a), while there was no difference between plants from open and shaded habitats from the South. There was also an overall significant interaction of region origin × habitat on rhizome biomass, but there were no significant differences between the Northern and Southern plants or plants from open and shaded habitats.

### Partitioning of assimilated ^14^C – effects of growing site

Plants grown in the South assimilated more ^14^C than plants in the North (8.8×10^5^ vs. 6.8×10^5^ DPM; *P*<0.001). When plants were placed in the South, they translocated a significantly higher amount of ^14^C to the rhizomes than in the North (*P*<0.001; Figure 3b). When clonal replicates of the same plants were grown in the North, they instead translocated a larger percentage of ^14^C to the shoots (*P*<0.001; Figure 3b). Translocation of ^14^C to the roots was constant between sites.

### Partitioning of assimilated ^14^C – effects of origin

Plants from shaded habitats tended to have more ^14^C in the rhizomes than plants from open habitats (habitat effect, *P* = 0.059; Table 3, Figure 4b). This was due to clones of the Shaded North origin translocating more ^14^C to rhizomes than plants from Open North habitats (*P*<0.05; Figure 4b). Within plants from shaded sites, clones of the Shaded North origin translocated more ^14^C to rhizomes than plants from Shaded South sites (*P* = 0.051; Figure 4b). Plants of the Open South origin did not differ from the other groups. The percentage of ^14^C in the roots did not differ between original regions (three-way ANOVA, ns).

## Discussion

All plants, both from Southern and Northern populations, grew larger at the Northern site and allocated more biomass to shoots. This contradicted our first hypothesis that plants would produce lower biomass at the Northern site. Our hypothesis was based on assumptions from classic plant-ecological studies, showing that plants had smaller stature as they followed altitudinal gradients, due to both a genetic and an environmental component to the plant's response [43]. In addition, plants experiencing range expansions northwards have lower biomass [11]–[13] and higher allocation to storage organs [14], [15]. In the present experiment, the *Aegopodium* plants grown in the North achieved higher biomass during the experimental period than the plants in the South. This is in line with studies showing that plants increase their relative growth rates at Northerly latitudes in order to compensate for the shorter growing season [15], [17], [18]. Day length is known to be a cue for plant growth [44]. In a physiological experiment with spinach, plants increased their growth when they were transferred from short days (8 hours sunlight) to long days (16 hours light) [45]. This was suggested to depend on increased production of gibberellins. The effect of placement (Site) in the present study could therefore be due to a physiological response to the longer days in Umeå during the experimental period. In addition, in a study of two forest herbs one species increased and another decreased in growth and reproduction along a latitudinal gradient, showing a difference in response to latitudinal gradients between species [20]. The response of rhizomatous plants to local conditions has also been shown to differ depending on the extent and longevity of its below-ground structures [46]. *Aegopodium podagraria* is an invasive weed typical of productive habitats. In this plant category, high relative growth rates enable efficient utilization of high resource availability, resulting in wide global distributions [21]. The higher allocation to shoots in the *Aegopodium* plants growing in the North might increase the utilization of light during the long days in the Northern growing season, resulting in higher biomass than in the South. The difference in biomass between genetically identical ramets placed in the North and the South demonstrated that there was strong phenotypic plasticity in *Aegopodium*. In addition, clones originating from the South and from the North differed in biomass production, suggesting genetically based differentiation between populations, which has previously been found among *Aegopodium* populations [35]. Contrary to our hypothesis, clones from North Sweden grew larger than plants from South Sweden at both experimental garden sites. The difference in biomass production between the Northern and Southern plants demonstrates that plants from the North were better able to produce large biomass, both under Southern and Northern conditions.

Plants of Northern habitats experience harsher conditions and have a longer time lag between carbohydrate assimilation and the time they are used for growth or reproduction than plants from Southern habitats. Both harsh conditions and a discrepancy between resource assimilation and usage have been suggested to lead to increased storage in clonal plants, and therefore higher translocation to storage was expected in the North [1], [9]. Opposite to what we expected, clones placed in the South had a higher rhizome biomass and allocated 40% more ^14^C to rhizomes than clones placed in the North. The harsh climate alone thus did not increase ^14^C - translocation to the rhizomes in *Aegopodium*. There was however higher ^14^C-translocation to the rhizomes in the clones from shaded, Northern habitats than in clones from open habitats, suggesting that integration patterns were a combined effect of region and habitat. In a previous experiment on *Aegopodium*, forest ramets depended more on resource sharing than garden ramets, suggesting that resource sharing was selected for in shaded habitats [5]. Although physiological integration has been identified as a trait of huge importance for clonal success [1], [2], [22], [47], it is poorly known which environmental factors that select for different degrees of physiological integration [2], [48]. Genotypes are thought to be able to vary in physiological integration, but relatively few studies have shown this [5], [49]–[52]. In the present experiment, higher translocation of ^14^C to the rhizomes was only found in the harsher, Northern habitats, showing that integration pattern can differ between habits, and that the interaction between regional climate and local habitat conditions (here light) was important.

Clonal plants that support ramets in unfavourable patches via physiological integration have been shown to be more invasive [2]. Plants like *Aegopodium*, which are strong growers and support ramets via physiological integration are therefore more likely to become invasive. For example, in the invasive clonal plant *Fallopia japonica*, investment of resources in the rhizomes and the efficient use of stored resources is suggested to contribute to the ability of this plant to establish in contrasting habitats [22], which is in line with what was found here for *Aegopodium*. At two contrasting sites along a latitudinal gradient, *Aegopodium* was shown to change its growth and carbon translocation pattern, supporting findings that high relative growth rates and physiological integration are traits that are important for the success of invasive clonal plants [2], [21]. The higher biomass at the Northern site was similar to that found in *Anemone nemorosa*, while *Milium effusum* in the same experiment declined along a latitudinal gradient [20]. The importance of stored resources as a buffer against temporal variability in resource availability was suggested by Suzuki and Stuefer [8], who also stated that little was known about if genetic differences in storage existed in natural populations of clonal plants. The emerging data from studies of populations of different origin show that clonal plants adapt to patterns of resource availability and heterogeneity, as well as to climatic conditions [5], [6], [49]–[51], [53]–[55]. Variation between genotypes from different habitats has been found for clonal traits such as clonal architecture [56], sexual vs. vegetative reproduction [54], carbon or phosphorus sharing [18], [49], [53] and competition between ramets [57]. Genets have also been demonstrated to be locally adapted to their environment in terms of morphology and reproduction [54] or to have environmentally based variation in physiological traits [51], [55]. In general, plants from resource-rich habitats, such as *Aegopodium*, have a higher maximum RGR or higher specific leaf area than plants from resource-poor habitats [58], [59]. In future experiments, RGR could be measured in order to investigate if plants adapted to the shorter growing periods in northern regions have a higher RGR than plants adapted to longer growing periods with shorter days (c.f. [13], [18]).The role of physiological integration and storage for the ability of clonal plants to establish in new habitats, or to persist when conditions change, needs further investigation and is a promising area for the understanding of invasiveness in clonal plants as well as for plants in general. To understand this, improved knowledge of how genetic variation in physiological integration is affected by regional climate and by local habitat conditions is needed.
